# Smurf1 regulation of DAB2IP controls cell proliferation and migration

**DOI:** 10.18632/oncotarget.8424

**Published:** 2016-03-27

**Authors:** Xiaoning Li, Xiangpeng Dai, Lixin Wan, Hiroyuki Inuzuka, Liankun Sun, Brian J. North

**Affiliations:** ^1^ Department of Pathophysiology, Basic Medical College, Jilin University, Changchun 130021, China; ^2^ Department of Pathology, Beth Israel Deaconess Medical Center, Harvard Medical School, Boston, MA 02215, USA

**Keywords:** DAB2IP, Smurf1, Akt, degradation, cancer

## Abstract

Tumor cell proliferation, survival and migration are regulated by the deletion of ovarian carcinoma 2/disabled homolog 2 (DOC-2/DAB2) interacting protein (DAB2IP), a tumor suppressor that serves as a scaffold protein for H-Ras and TRAF2. Importantly, the oncogenic histone methyl-transferase EZH2 epigenetically down-regulates DAB2IP in a variety of tumors. Recently, we demonstrated that DAB2IP is negatively regulated by Akt-dependent phosphorylation and SCF^Fbw7^-mediated degradation. Here, we further identify the oncoprotein Smurf1, an E3-ubiquitin ligase, as a novel negative regulator of DAB2IP. Smurf1-mediated cellular proliferation and migration are largely dependent on the presence of DAB2IP, suggesting that DAB2IP is a key effector molecule of Smurf1 oncogenic function. Additionally, we identify that similar to DAB2IP, Smurf1 is also a target of phosphorylation by both Akt1 and Akt2 kinases, which enhances Smurf1 abundance, leading to a reduction in DAB2IP. Given the role of DAB2IP in tumorigenesis and metastasis, our data identify Smurf1 as an upstream oncogenic factor that negatively regulates DAB2IP to govern aberrant cell growth and migration.

## INTRODUCTION

Tumor metastasis is a major obstacle to curing cancer and is a driving mechanism to increased mortality in cancer patients [1]. For many types of cancer, tumor cells gain the ability to migrate to distant organs, which ultimately leads to organ failure and death [1, 2]. Elucidating the underlying molecular mechanisms that promote tumor growth and metastasis will provide further insight for the development of therapeutics, in part by eliminating metastatic cancer cells. While the molecular mechanisms remain poorly defined, overexpression of specific oncoproteins [3] or downregulation of specific tumor suppressor proteins [4] have been shown to play important roles in tumor growth and metastasis. To this end, deletion of ovarian carcinoma 2/disabled homolog 2 (DOC-2/DAB2) interacting protein (DAB2IP), is a tumor suppressor in various types of human cancer [5–9] where loss of DAB2IP expression is associated with poor prognosis and increased tumor metastasis [6, 8–11].

DAB2IP is frequently downregulated by epigenetic modification in multiple aggressive cancers. Specifically, in prostate cancer, DAB2IP expression is repressed by promoter methylation and histone modification, primarily through the action of the histone methyltransferase EZH2 [12, 13], whereas in breast cancer [6], lung cancer [8], and gastrointestinal tumors [14], aberrant promoter hypermethylation was also shown to downregulate DAB2IP. In prostate cancer, it was identified that downregulation of DAB2IP expression promotes resistance to ionizing radiation [15], initiates epithelial-to-mesenchymal transition [11] and drives tumor growth and metastasis [16]. Furthermore, DAB2IP is involved in TNFα-induced apoptosis in prostate cancer cells in part by suppressing the ASK1-JNK and PI3K-AKT pathway [11], and in endothelial cells via inhibiting the ASK1-JNK pathway [17].

Through interaction with various factors, DAB2IP can modulate the activities of various pathways including Ras-Raf-ERK, ASK1-JNK, and PI3K-Akt, through which loss of DAB2IP can further deregulate survival and apoptosis pathways, leading to tumor development. DAB2IP primarily functions as a Ras GTPase-activating protein (RasGAP) to accelerate GTP hydrolysis of Ras proteins, thus reducing abundance of active GTP-loaded Ras, which suppresses the RAS/RAF/MEK/ERK signaling cascade [18], regulates the ASK1 pathway by blocking interaction of ASK1 with its inhibitor 14-3-3 [17], binds to and inactivates the Akt kinase [19], and regulates the NF-κB pathway through bidning to Traf2 [16]. We previously identified that DAB2IP is negatively regulated by both Akt and Fbw7 [20]. Akt1 can phosphorylate DAB2IP on S847, which regulates interaction between DAB2IP and its effector molecules H-Ras and Traf2. Additionally, DAB2IP can be degraded through the ubiquitin-proteasome pathway by SCF^Fbw7^. DAB2IP harbors two Fbw7 phosho-degron motifs that can be regulated by the kinase CK1δ to promote its degradation by SCF^Fbw7^. These data suggest that DAB2IP transduces multiple upstream signaling events to control cellular growth and migration. Thus, it is critically important to further understand the exact upstream regulatory pathways that control DAB2IP to develop effective anti-metastasis therapeutics.

The HECT domain-containing E3 ubiquitin ligase Smurf1 (SMAD ubiquitylation regulatory factor-1) functions to regulate the BMP (bone morphogenetic protein) pathway through targeting SMAD proteins for degradation [21]. Unlike other members of the NEDD4 family of E3 ligases which are regulated through an auto-inhibitory mechanism mediated by intramolecular interactions [22], Smurf1 lacks this auto-inhibitory intra-molecular interaction [23]. However, Smurf1 appears to be precisely regulated by multiple pathways, including activation of Smurf1 by CKIP1 [24], direct neddylation [25], and negative regulation of Smurf1 by the E3 ubiquitin ligases Fbxl15 [26] and Fbxo3 [27]. In regard to the activation of Smurf1 via neddylation, increased expression of Smurf1 and Nedd8 in colorectal cancer correlates with robust cancer progression and poor prognosis [25]. It has been recently reported that Smurf1 is regulated by another E3 ligase, Cdh1, through a mechanism by which Cdh1 disrupts Smurf1 homodimers in an E3 ligase-independent manner [28]. Under conditions where Cdh1 is either depleted or inactivated, reduced Smurf1 can drive a number of downstream pathways including osteoblast differentiation through MEKK2 activation [28].

While Smurfs (Smurf1 and Smurf2) were initially identified as regulators of TGF-β/BMP signaling, identification of Smurf substrates has also implicated Smurfs in diverse cellular processes including cell-cycle progression, cell proliferation, differentiation, DNA damage response, maintenance of genomic stability, and metastasis [29]. For example, Smurf1 was found to promote cell migration by targeting RhoA for ubiquitination-mediated proteolysis [30]. Given the role of Smurf1 in regulating metastasis, we set out to determine if Smurf1 controls metastasis by regulating DAB2IP stability. Here, we identified that Smurf1 degrades DAB2IP and furthermore, that the effects on migration and cell proliferation observed by loss of Smurf1 are due in part to upregulation of DAB2IP.

## RESULTS

### Smurf1 interacts with and promotes ubiquitination-dependent degradation of DAB2IP

**Figure 1:**
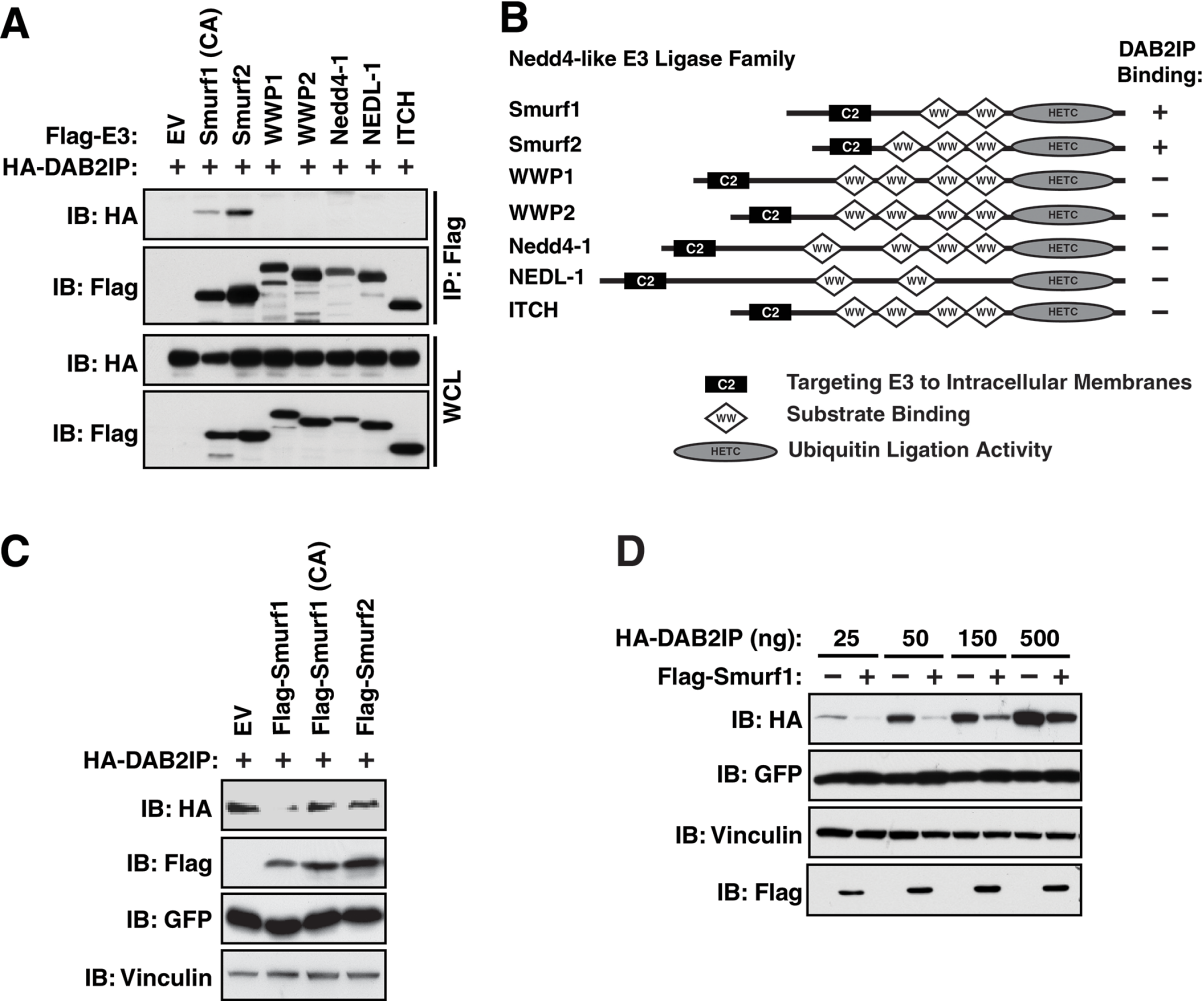
Interaction of DAB2IP with Smurf1 regulates DAB2IP protein abundance (**A**) 293T cells were transfected with Flag-tagged Nedd4-like E3 ligases and HA-DAB2IP were immunoprecipitated with anti-Flag, and western blotted with antibodies against Flag and HA. (**B**) Schematic representation of domain structures of Nedd4-like E3 ligases. (**C**) 293T cells were transfected with Flag-Smurf1 (WT or a catalytically inactive C725A mutant) or Smurf2 and HA-DAB2IP. Whole cell lysates were prepared and western blotted with antibodies against HA, Flag, GFP, and Tubulin. (**D**) 293T cells were transfected with HA-DAB2IP with or without Flag-Smurf1. Whole cell lysates were prepared and western blotted with antibodies against HA, Flag, GFP, and Tubulin.

Given that DAB2IP primarily functions as a tumor suppressor, we postulated that Nedd4-like E3 ligases, which largely function as oncoproteins, may regulate DAB2IP due to their shared membrane localization [21], which would provide a unique regulatory mechanism of DAB2IP protein stability. To determine if DAB2IP interacted with any of the Nedd4-like E3 ligases, we co-expressed DAB2IP with Smurf1, Smurf2, WWP1, WWP2, Nedd4-1, NEDL-1 and ITCH in 293T cells which were treated with MG132 to block 26S proteasome-mediate proteolysis. Notably, we identified that DAB2IP specifically interacts with both Smurf1 and Smurf2 (Figure 1A–1B). To assess if DAB2IP was degraded in response to co-expression with Smurf1 and Smurf2, we co-transfected Smurf1 wild-type, as well as a activity-defective version of Smurf1 in which the cysteine residue in the active site is replaced with an alanine (C725A) [28], and Smurf2, and assessed the steady state protein levels of HA-DAB2IP. We observed that ectopic expression of Smurf1 reduced protein levels of DAB2IP in an E3 ligase activity-dependent manner whereas Smurf2 was unable to reduce protein levels of DAB2IP (Figure 1C–1D). These studies demonstrate that overexpression of Smurf1 can control the protein abundance of exogenous DAB2IP, indicating DAB2IP as a potential ubiquitin substrate of Smurf1.

**Figure 2:**
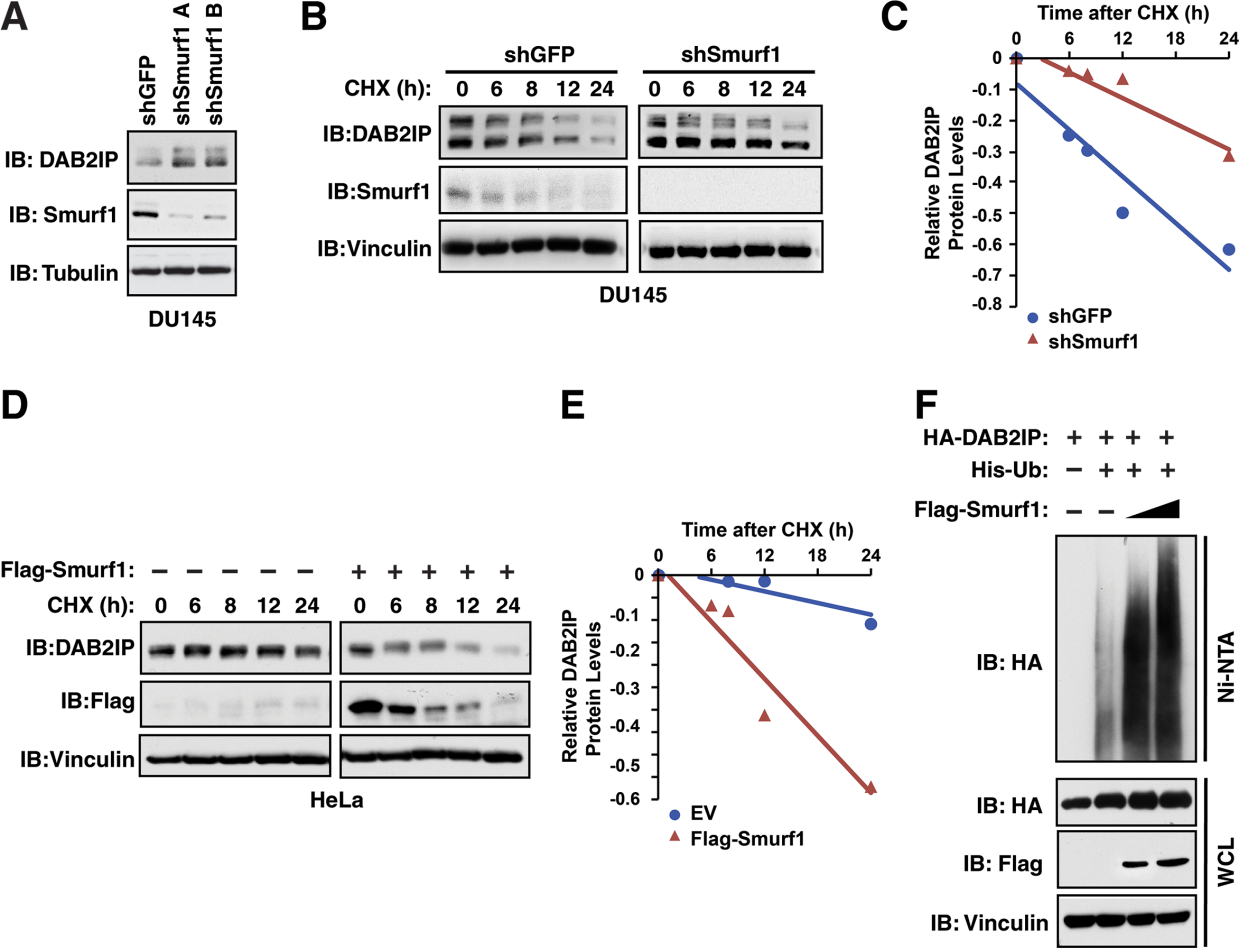
Smurf1 regulates DAB2IP protein stability via ubiquitination (**A**) DU145 cells were infected with virus expressing shRNA against GFP and Smurf1. Following selection of infected cells, whole cell lysates were prepared and western blotted with antibodies against Smurf1, DAB2IP and Tubulin. (**B**) DU145 cells were infected with virus expressing shRNA against GFP and Smurf1. Following selection of infected cells, cells were treated for indicated times with cycloheximide (CHX). Whole cell lysates were prepared and western blotted with antibodies against Smurf1, DAB2IP and Vinculin. (**C**) Quantification of western blots shown in B. (**D**) HeLa cells were transfected with control vector or Flag-Smurf1 and treated for indicated times with cycloheximide (CHX). Whole cell lysates were prepared and western blotted with antibodies against Flag, DAB2IP and Vinculin. (**E**) Quantification of western blots shown in D. (**F**) 293T cells transfected with HA-DAB2IP, His-Ubiquitin and increasing concentrations of Flag-Smurf1 were treated with MG132 for 16 hours. His-Ubiquitinated proteins were purified with Ni-NTA and eluates along with whole cell extracts were western blotted HA, Flag and Vinculin.

To further examine whether DAB2IP protein abundance is under the control of Smurf1, we assessed the levels of endogenous DAB2IP following depletion of Smurf1. Utilizing two independent shRNAs targeting Smurf1, we observed that depletion of Smurf1 resulted in an increase in the abundance of endogenous DAB2IP in DU145 cells (Figure 2A). This effect of depletion of Smurf1 on DAB2IP was also observed in T98G and MCF7 cells (Supplementary Figure S1A–S1B). Furthermore, this increased abundance in DAB2IP in response to depletion of Smurf1 was due to an increase in protein half-life (Figure 2B–2C). Conversely, the half-life of DAB2IP in HeLa cells was dramatically shortened in cells overexpressing Smurf1 (Figure 2D–2E). Finally, given that Smurf1 is an E3 ubiquitin ligase, we determined if Smurf1 controls DAB2IP protein abundance through regulation of DAB2IP ubiquitination. Upon expression of Smurf1 in 293T cells, we observed a robust, dose-dependent, increase of DAB2IP ubiquitination in response to Smurf1 overexpression (Figure 2F). These results in combination indicate that Smurf1 controls the protein stability of DAB2IP through regulation of DAB2IP ubiquitination levels.

**Figure 3:**
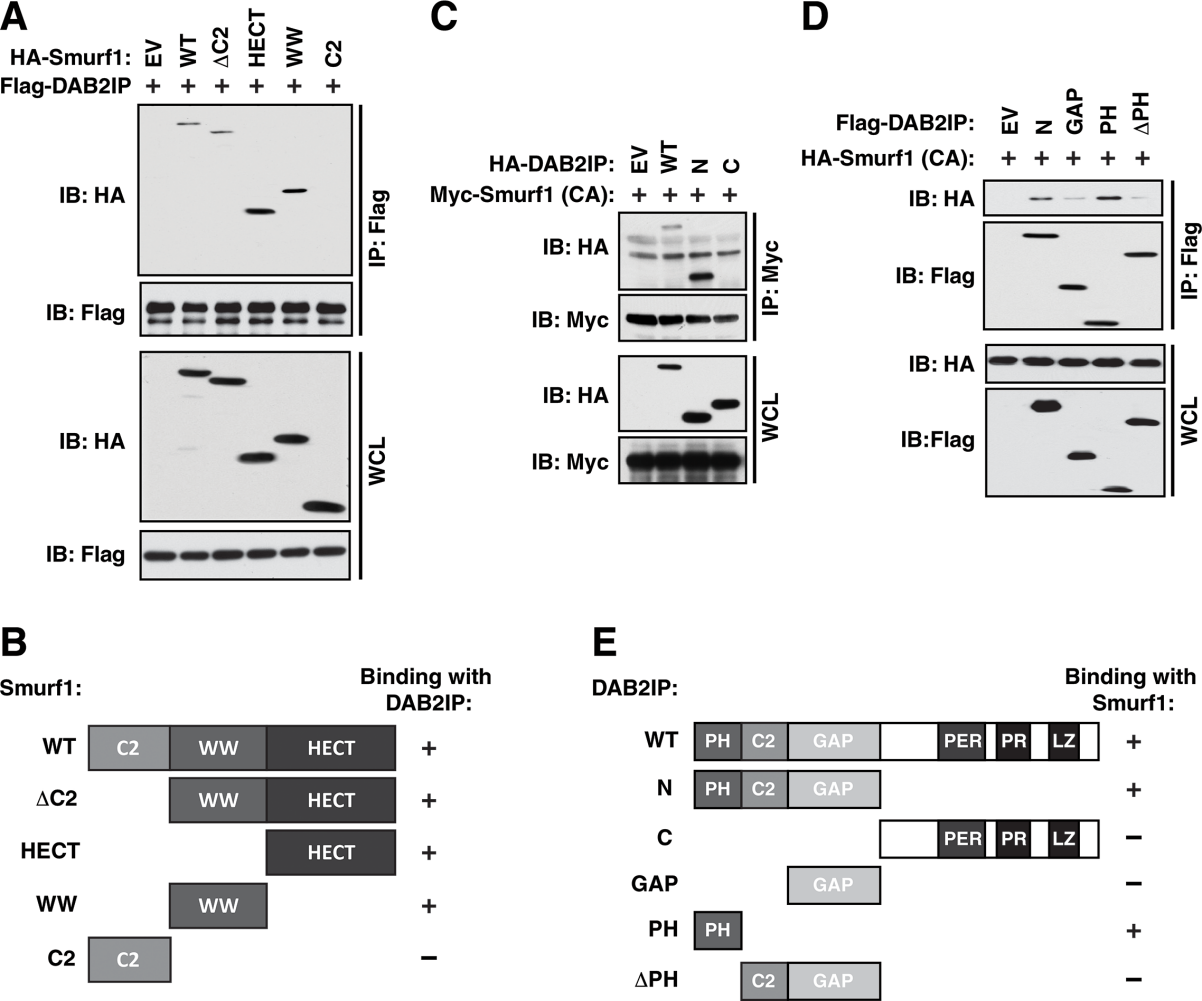
Mapping the interaction between Smurf1 and DAB2IP (**A**) 293T cells transfected with full-length and truncation mutants of Flag-Smurf1 with HA-DAB2IP were immunoprecipitated with anti-Flag, and western blotted with antibodies against Flag and HA. (**B**) Schematic diagram of Smurf1 truncations utilized in A showing domain structures present in each truncation construct and their interaction ability with DAB2IP. (**C**) 293T cells transfected with HA-DAB2IP wild-type and truncation mutants with full-length Myc-Smurf1 C725A (CA) were immunoprecipitated with anti-Myc, and western blotted with antibodies against Myc and HA. (**D**) 293T cells transfected with N-terminal HA-DAB2IP and further truncation mutants of the DAB2IP N-terminus with full-length HA-Smurf1 C725A (CA) were immunoprecipitated with anti-Flag and western blotted with antibodies against Flag and HA. (**E**) Schematic diagram of DAB2IP truncations utilized in C and D showing domain structures present in each truncation construct and their interaction ability with Smurf1.

To further elucidate the molecular mechanisms by which Smurf1 controls DAB2IP ubiquitination and degradation, we set out to map the interaction domains in both Smurf1 and DAB2IP. Similar to other Nedd4-like E3 ligases, Smurf1 is composed of three main functional domains, a membrane interacting C2 domain, a substrate binding WW domain and a catalytic HECT domain [22] (Figure 1B). To map which domain(s) in Smurf1 are necessary for interaction with DAB2IP, we created 4 deletion mutants of Smurf1, ΔC2 which lacks the C2 domain, and each domain individually (HECT, WW, and C2). Co-expressing these mutants with DAB2IP in 293T cells, followed by co-immunoprecipitation analysis, we identified that both the WW and HECT domains in Smurf1 are largely responsible for the interaction with DAB2IP (Figure 3A–3B). To define the specific domains within DAB2IP responsible for its interaction with Smurf1 we generated DAB2IP proteins that deleted either the C terminal or N-terminal halves of the protein. Co-expressing these mutants with Smurf1 in 293T cells, followed by co-immunoprecipitation analysis, we identified that the N-terminal half of DAB2IP is responsible for binding to Smurf1 (Figure 3C and 3E). The N-terminal domain of DAB2IP contains three important regulatory domains, the PH, C2, and GAP domains. We observed that further deletion of the PH and C2 (ΔPHC2) or the PH domain alone (ΔPH) largely abolished interaction, whereas expressing the PH domain alone was sufficient to promote interaction with Smurf1 to a similar level as the intact N-terminal domain (Figure 3D–3E).

### Akt-mediated phosphorylation of DAB2IP does not influence its ubiquitination-dependent degradation by Smurf1

**Figure 4:**
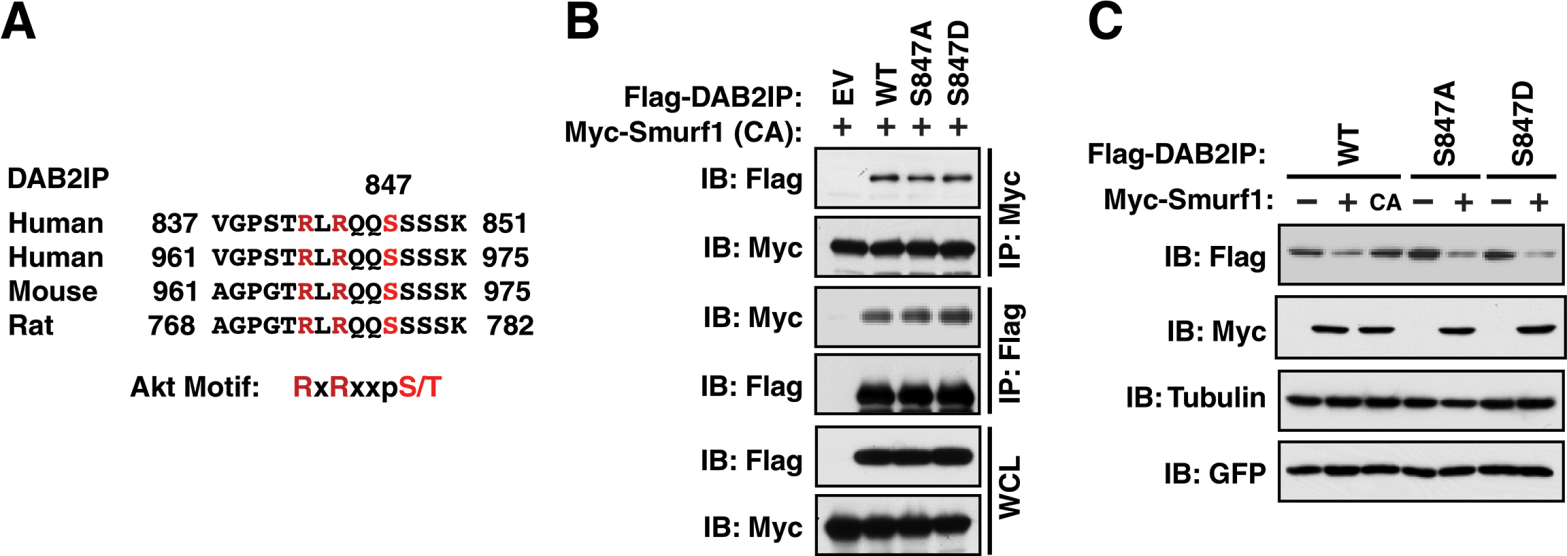
DAB2IP phosphorylation by Akt does not promote association with Smurf1 (**A**) Alignment of Akt phosphorylation sites in human, mouse and rat DAB2IP. (**B**) 293T cells transfected with wild-type and Akt phosphorylation site mimetic (S847D) and non-phosphorylatable (S847A) mutants of Flag-DAB2IP with Myc-Smurf1 were immunoprecipitated with either anti-Flag or anti-Myc, and western blotted with antibodies against Myc and Flag. (**C**) 293T cells were transfected with wild-type and Akt phosphorylation site mimetic (S847D) and non-phosphorylatable (S847A) mutants of Flag-DAB2IP with or without Myc-tagged Smurf1. Whole cell lysates were prepared and western blotted with antibodies against HA, Myc, GFP, and Tubulin.

Previously we identified that degradation of DAB2IP by Fbw7 was carried out in conjunction with phosphorylation by Akt at Serine-847 in DAB2IP (Figure 4A) [20]. While S847 does not lie within the Smurf1 interaction domain on DAB2IP, we wanted to further determine if the phosphorylation status of DAB2IP at this Akt site was important for the interaction between DAB2IP and Smurf1. Utilizing phosphorylation defective (S847A) and phosphorylation mimetic (S847D) mutants of DAB2IP, we assessed if these mutants modulated the interaction between DAB2IP and Smurf1 or the degradation of DAB2IP by Smurf1. Notably, we found that the phosphorylation of DAB2IP was neither important for the interaction between these two factors (Figure 4B), nor was it important for the degradation of DAB2IP by Smurf1 (Figure 4C). These data indicate that while Smurf1 degrades DAB2IP, it does so independently of the mechanisms utilized to control DAB2IP by Fbw7, highlighting this pathway as a novel regulatory mechanism controlling DAB2IP protein abundance.

### Akt could affect DAB2IP stability in part by stabilizing Smurf1

**Figure 5:**
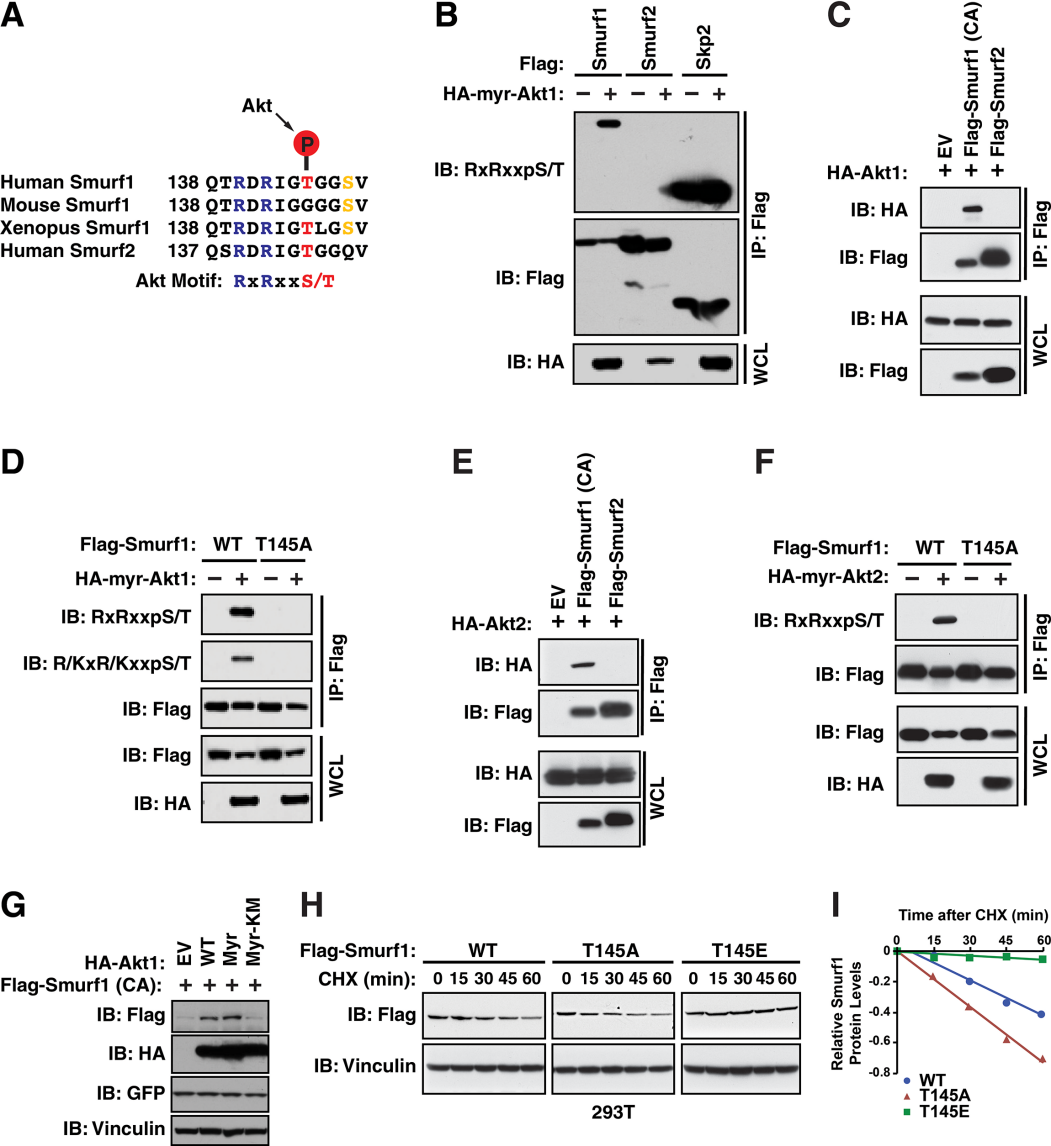
Smurf1 is a target of Akt (**A**) Alignment of human, mouse, and xenopus Smurf1 and human Smurf2 sequences surrounding putative Akt substrate motif. (**B**) 293T cells transfected with Flag-Smurf1, Flag-Smurf2 and Flag-Skp2 with or without HA-Myr-Akt1 were immunoprecipitated with anti-Flag, and western blotted with antibodies against RxRxxpS/T, Flag, and HA. (**C**) 293T cells transfected with HA-Myr-Akt1 and either Flag-Smurf1 C725A (CA) or Flag-Smurf2 were immunoprecipitated with anti-Flag, and western blotted with antibodies against Flag and HA. (**D**) 293T cells transfected with wild-type or T145A mutant Flag-Smurf1, with or without HA-Myr-Akt1 were immunoprecipitated with anti-Flag, and western blotted with antibodies against R/KxR/KxxpS/T, Flag, and HA. (**E**) 293T cells transfected with HA-Myr-Akt2 and either Flag-Smurf1 C725A (CA) or Flag-Smurf2 were immunoprecipitated with anti-Flag, and western blotted with antibodies against Flag and HA. (**F**) 293T cells transfected with wild-type or T145A mutant Flag-Smurf1, with or without HA-Myr-Akt2 were immunoprecipitated with anti-Flag, and western blotted with antibodies against RxRxxpS/T, Flag, and HA. (**G**) 293T cells were transfected with Flag-Smurf1 C725A with either vector, wild-type Akt1, Myr-Akt1, or Myr-Akt1 KM (catalytically inactive mutant). Whole cell lysates were prepared and western blotted with Flag, HA, GFP, Vinculin. (**H**) 293T cells transfected with wild-type, T145A, or T145E Flag-Smurf1 and treated for indicated times with cycloheximide (CHX). Whole cell lysates were prepared and western blotted with antibodies against Flag and Vinculin. (**I**) Quantification of western blots shown in H.

While phosphorylation of DAB2IP by Akt was not important for its regulation by Smurf1, we identified that Smurf1 also contains an Akt phosphorylation consensus motif at Threonine 145 (Figure 5A). To assess if Smurf1 is a functional Akt substrate, we co-expressed myristylated, constitutively active, form of Akt1 (Myr-Akt1) with Smurf1 and Smurf2. We found, using an Akt1 substrate antibody, which recognizes the RxRxxpS/T Akt phosphorylation motif, that Smurf1 phosphorylation was induced following co-expression of Myr-Akt1, whereas Smurf2 was not, even though it also harbors an Akt1 consensus sequence (Figure 5B). One potential mechanism driving specificity of Akt1-mediated phosphorylation towards Smurf1, is that Smurf1 is able to interact with Akt1, while Smurf2 does not interact with Akt1 in cells (Figure 5C). Furthermore, phosphorylation of Smurf1 by Akt1 was carried out specifically on the T145 residue, as mutation of this site abolished recognition of Smurf1 by the Akt1 phosphorylation motif antibody (Figure 5D). These results indicate that Smurf1, but not Smurf2, is targeted by Akt1 for phosphorylation. Similar to Akt1, we also observed that Akt2 interacts with, and phosphorylates, Smurf1, but not Smurf2 (Figure 5E–5F).

To determine if phosphorylation of Smurf1 by Akt is important for its regulation of DAB2IP, we utilized phosphorylation-defective (T145A) and phosphorylation-mimetic (T145E) mutants of Smurf1 and assessed if these mutants modulated the ability of Smurf1 to interact and/or degrade DAB2IP. We found that phosphorylation of Smurf1 at T145 was neither important for its interaction with DAB2IP (Supplementary Figure S2A), nor was it important for the degradation of DAB2IP by Smurf1 (Supplementary Figure S2B). Furthermore, utilizing these mutants of Smurf1, we also found that Akt-mediated phosphorylation of Smurf1 was not important for its regulation of Smad1, another previously characterized ubiquitin substrate of Smurf1 (Supplementary Figure S2C) [31].

To determine if Akt-mediated phosphorylation of Smurf1 regulated Smurf1 abundance, overexpression of either wild-type or myristoylated Akt1 led to an increase in Smurf1 protein abundance, which was dependent on Akt1 activity as a kinase-dead mutant (Myr-Akt1 KM) did not stabilize Smurf1 (Figure 5G). Given that Akt-mediated phosphorylation occurs on T145, we tested if mutation of T145 to either a non-phosphorylatable alanine (T145A) or to a phospho-mimetic (T145E) influenced Smurf1 stability. Consistent with a role for phosphorylation at T145 by Akt resulting in stabilization of Smurf1, we found that the Smurf1-T145E mutant had an increased half-life compared to wild-type Smurf1, and a non-phosphorylatable T145A had a shorter half-life than both T145E or wild-type Smurf1 (Figure 5H–5I). These data indicate that Smurf1 is under the control of Akt-mediated phosphorylation that regulates Smurf1 protein stability.

**Figure 6:**
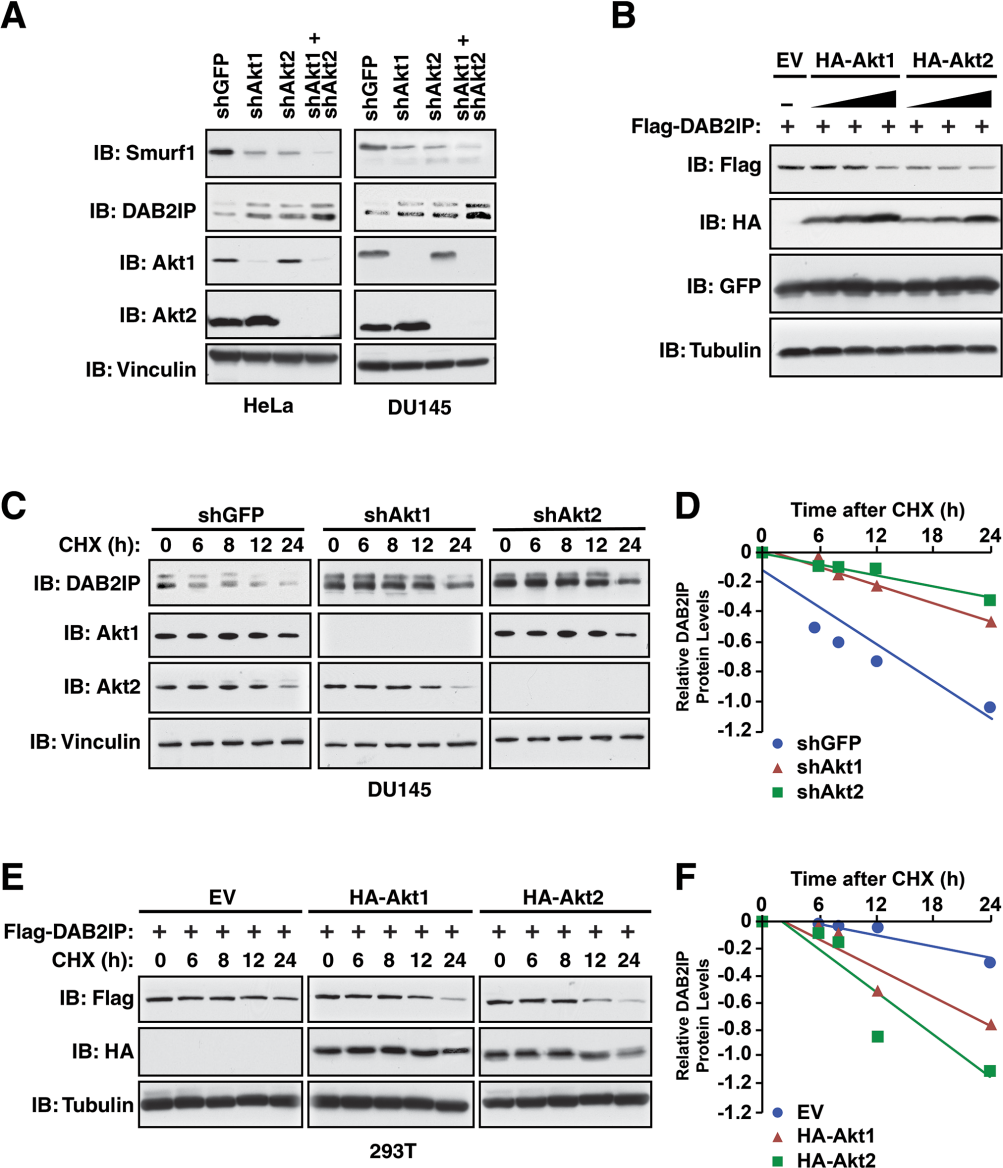
Control of DAB2IP stability by Akt1/Smurf1 degradation cascade (**A**) HeLa and DU145 cells were infected with virus expressing shRNA against GFP, Akt1, Akt2, or co-infected with virus expressing shRNA against Akt1 and Akt2. Following selection of infected cells, whole cell lysates were prepared and western blotted with antibodies against Smurf1, DAB2IP, Akt1, Akt2 and Vinculin. (**B**) 293T cells transfected with Flag-DAB2IP and increasing concentrations of HA-Akt1 or HA-Akt2 along with GFP (transfection control). Whole cell lysates were prepared and western blotted with antibodies against Flag, HA, GFP and Tubulin. (**C**) DU145 cells were infected with virus expressing shRNA against GFP, Akt1, or Akt2. Following selection of infected cells, cells were treated for indicated times with cycloheximide (CHX). Whole cell lysates were prepared and western blotted with antibodies against DAB2IP, Akt1, Akt2 and Vinculin. (**D**) Quantification of western blots shown in C. (**E**) 293T cells transfected with Flag-DAB2IP and increasing concentrations of HA-Akt1 or HA-Akt2. Cells were treated for indicated times with cycloheximide (CHX). Whole cell lysates were prepared and western blotted with antibodies against Flag, HA and Tubulin. (**F**) Quantification of western blots shown in E.

We previously identified the Akt pathway as an upstream regulator of the DAB2IP tumor suppressor protein, thus we wanted to next assess if Smurf1 phosphorylation by Akt has any role in Akt-mediated regulation of DAB2IP. Notably, depletion of either Akt1 or Akt2 in HeLa or DU145 cells led to a significant reduction in Smurf1 protein (Figure 6A), suggesting that phosphorylation of Smurf1 by either Akt1 or Akt2 promoted Smurf1 stability. Reduction in Smurf1 following depletion of Akt1 or Akt2 also resulted in an increase in DAB2IP (Figure 6A), consistent with a role for Smurf1 in regulation DAB2IP stability. Interestingly, we also observed a more pronounced reduction in Smurf1 protein and a greater subsequent induction of DAB2IP following the co-depletion of both Akt1 and Akt2 than with depletion of either Akt isoform alone (Figure 6A). Consistent with this result, we found that increasing Akt1 or Akt2 activity resulted in a concomitant dose-dependent decrease in DAB2IP protein abundance (Figure 6B). These data highlight that the protein abundance of the E3 ubiquitin ligase Smurf1 is controlled by Akt-mediated phosphorylation that subsequently results in alteration in protein abundance of Smurf1 substrates such as DAB2IP. Thus, the Akt oncogenic signaling pathway could negatively regulate DAB2IP through both Akt-mediated direct phosphorylation of DAB2IP to reduce its binding with K-Ras, as well as through promoting Smurf1 stability to increase DAB2IP degradation. To test a role for Akt-mediated phosphorylation in regulating DAB2IP stability, we measured the half-lives of DAB2IP following depletion of either Akt1 or Akt2. We observed that knocking down either Akt isoforms led to an increase in DAB2IP protein stability compared with shGFP control (Figure 6C–6D). Consistent with a role for Akt isoforms in regulating DAB2IP, we observed that overexpression of Akt1 or Akt2 lead to a decrease in DAB2IP protein stability (Figure 6E–6F). Together these results suggest that Akt-mediated phosphorylation of Smurf1 regulates Smurf1 stability, which indirectly controls DAB2IP protein abundance, suggesting that increased Akt activity during tumorigenesis may impact cellular growth and migration in a DAB2IP-dependent manner.

### The Akt/Smurf1 signaling axis promotes cell growth and migration largely by promoting DAB2IP degradation

**Figure 7:**
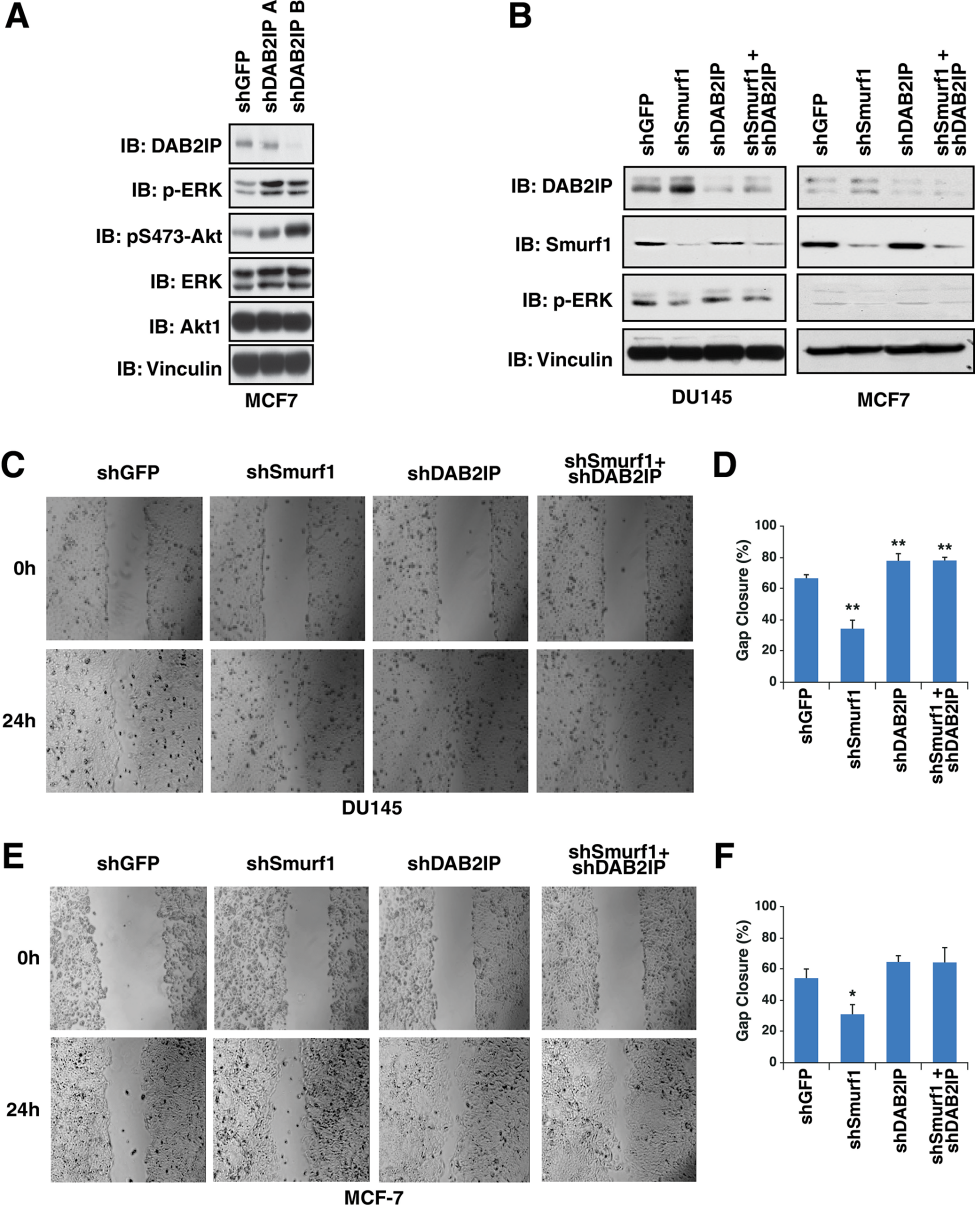
Control of migration by Smurf1 is dependent on DAB2IP (**A**) MCF-7 cells were infected with virus expressing shRNA against GFP and DAB2IP. Following selection of infected cells, whole cell lysates were prepared and western blotted with antibodies against DAB2IP, pERK, ERK, pS473-Akt1, Akt1, and Vinculin. (**B**) DU145 and MCF-7 cells were infected with virus expressing shRNA against GFP, Smurf1, DAB2IP or Smurf1 and DAB2IP together. Following selection of infected cells, lysates were western blotted with antibodies against Smurf1, DAB2IP, pERK, and Vinculin. (**C**) *In vitro* scratch assay at 0 and 24 hours using DU145 cells described in B. (**D**) Quantification of gap closure of *in vitro* scratch assay described in C. (**E**) *In vitro* scratch assay at 0 and 24 hours using MCF-7 cells described in B. (**F**) Quantification of gap closure of *in vitro* scratch assay described in E. Error bars represent standard deviation. *p < 0.05, **p < 0.005.

Previously, DAB2IP has been shown to be important for inhibiting both tumorigenesis and metastasis [6, 8–11], whereas Smurf1 has been shown to be a potent oncogene. Therefore we set out to determine whether these tumor regulatory mechanisms mediated by Smurf1 are dependent on DAB2IP. Previous studies have demonstrated that loss of DAB2IP induces MAPK signaling where overexpression of DAB2IP suppresses MAPK signaling [18]. Consistent with these previous studies, we demonstrate that loss of DAB2IP led to an induction of pERK and pAKT levels (Figure 7A). As expected, depletion of Smurf1 led to an increase in DAB2IP and subsequent reduction in pERK levels (Figure 7B). However, knockdown of DAB2IP in combination with depletion of Smurf1 abrogated the observed changes in pERK that is induced by depletion of Smurf1 alone (Figure 7B), arguing that Smurf1 utilizes the DAB2IP signaling pathway to govern ERK signaling strength.

To further determine if DAB2IP induction due to depletion of Smurf1 was necessary for the effect of Smurf1 on cell proliferation and migration, we assessed cell migration and proliferation following depletion of Smurf1 or DAB2IP individually or in combination. To test cell migration, we carried out cell scratch assays and measured cellular migration into the gap 24 hours post scrapping. We found that cells depleted of Smurf1 had reduced migration, whereas those depleted of DAB2IP migrated faster than control cells (Figure 7C–7F). Consistent with our results above, co-depletion of both DAB2IP and Smurf1 blocked the effects of knockdown of Smurf1, indicating that the increase in DAB2IP protein abundance following loss of Smurf1 was critically important for the effect of Smurf1 on cellular migration (Figure 7C–7F).

**Figure 8:**
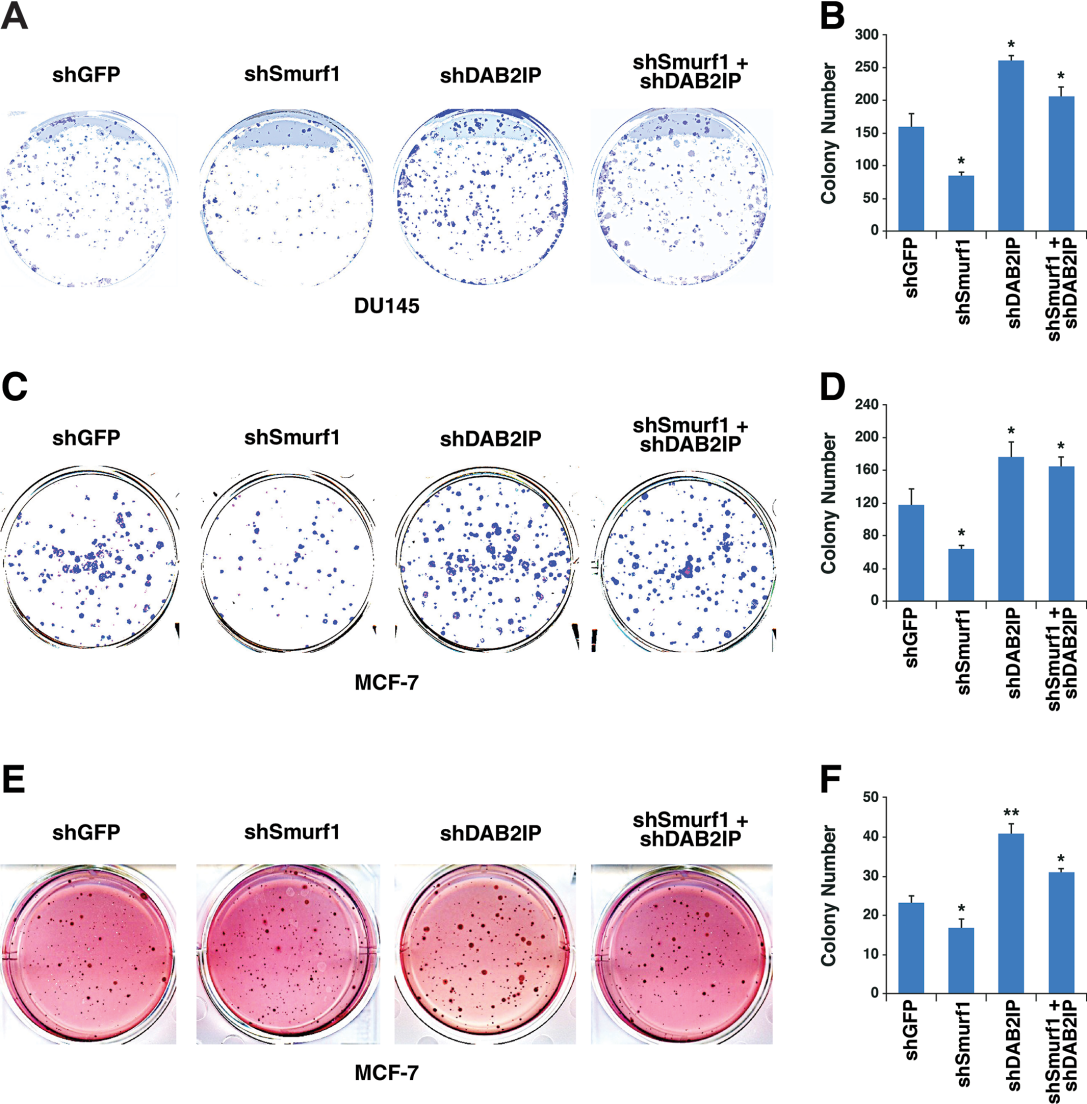
Control of cellular proliferation by Smurf1 is dependent on DAB2IP (**A**) Colony formation assay using DU145 cells described in Figure 7B. (**B**) Quantification of colony number of colony formation assays described in A. (**C**) Colony formation assay using MCF-7 cells described in Figure 7B. (**D**) Quantification of colony number of colony formation assays described in C. (**E**) Soft agar assay using MCF-7 cells described in Figure 7B. (**F**) Quantification of colony number of soft agar assays described in E. Error bars represent standard deviation. *p < 0.05, **p < 0.005.

Similar to what we observed in a cellular migration assay, we measured cell proliferation utilizing both a colony formation assay (Figure 8A–8D) as well as a soft agar assay (Figure 8E–8F), and found that depletion of Smurf1 alone reduced colony growth, which was dependent on the presence of DAB2IP, as further depletion of DAB2IP blocked the effect of Smurf1 knockdown on cell proliferation. These results together support the model that the Akt/Smurf1 oncogenic signaling pathway promotes cellular proliferation and migration largely through degrading the DAB2IP tumor suppressor protein.

## DISCUSSION

**Figure 9:**
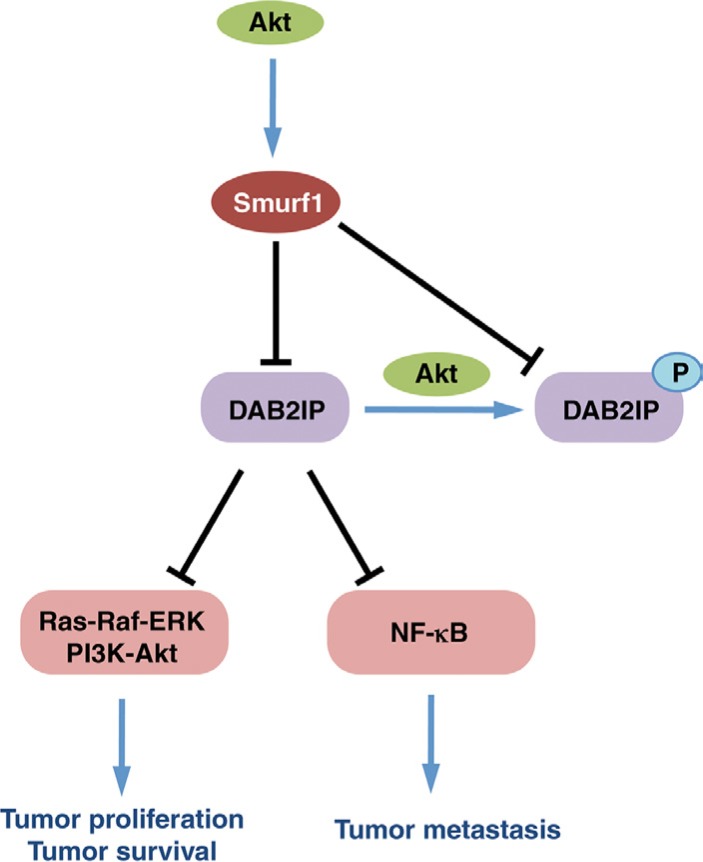
Schematic representation of control of DAB2IP by Smurf1 and Akt1

Here we have elucidated a novel mechanism controlling DAB2IP stability mediated by the Akt/Smurf1 oncogenic signaling pathway. Our results indicate that DAB2IP interacts specifically with Smurf1 and Smurf2 of the Nedd4-like E3 ligase family, and that Smurf1 is largely responsible for degradation of DAB2IP through ubiquitination-mediated proteolysis. Consequently, DAB2IP regulation by Smurf1 is intimately linked to the ability of Smurf1 to control both cellular proliferation as well as migration, likely through the modulation of downstream Ras-MAPK and NF-κB signaling pathways (Figure 9).

Importantly, our previous study reported that DAB2IP was functionally downregulated by both SCF^Fbw7^-mediated degradation and Akt-mediated phosphorylation to disrupt the interaction of DAB2IP and K-Ras as means to reduce its suppressive role towards the Ras/pERK signaling axis [20]. Our results presented here indicate that Smurf1 regulation of DAB2IP appears to function independently of those pathways. Of particular interest in light of these results here and the results of our previous study [20] is how these two E3 ligases, Smurf1 and SCF^Fbw7^, are controlling DAB2IP degradation, whether each is working in different cell types, or if there is a temporal or spatial specificity to the control of DAB2IP by each of these E3 ligase complexes.

We also identified that Smurf1 itself is targeted for phosphorylation by Akt1 and Akt2, regulating its stability. Interestingly, even though both Akt1 and Akt2 appear to phosphorylate the same amino acid (T145), they appear to do so in a partially non-redundant fashion, however further studies are necessary to fully delineate the cell specificity as well as potential temporal and spatial regulation of Smurf1 by the Akt1 and Akt2 kinases. As a result, depleting Akt resulted in reduced Smurf1 abundance and consequently elevated DAB2IP activity. As Akt kinase activity is frequently elevated in various types of human cancer, our results suggest that in these pathological conditions elevated Akt activity could potently reduce the abundance of the DAB2IP tumor suppressor protein through control of Smurf1 abundance, conferring growth advantage or metastatic ability in these tumor cells. Therefore based on these results and our previous study [20], we believe that the Akt pathway may coordinately target DAB2IP through two distinct mechanisms: indirectly through Akt-mediated stabilization of Smurf1 leading to increased degradation of DAB2IP (this study), and Akt-mediated functional inactivation of DAB2IP by direct phosphorylation [20]. Two recent studies have also demonstrated that Smurf1 is targeted for degradation by two E3 ubiquitin ligases, Fbxl15 [26] and Fbxo3 [27]. It is interesting to speculate that T145 phosphorylation by Akt1 and Akt2 may form part of a phospho-degron motif in Smurf1 for recognition by one or both of these E3 ubiquitin ligases.

Therefore, our results shed new light on the molecular mechanisms by which Smurf1 drives tumorigenesis and metastasis. By targeting DAB2IP, Smurf1 has direct control over the Ras-MAPK and NF-κB oncogenic pathways. Through governing these pathways, Smurf1 can drive both tumorigenesis as well as metastasis. Thus identifying mechanisms to modulate the Smurf1 E3 ligase activity could provide a novel therapeutic avenue for cancer by targeting both early phases of tumorigenesis and later stages of metastasis, thereby attacking cancer in two phases and enhancing cancer patient survival.

## MATERIALS AND METHODS

### Cell culture

HeLa, 293T, T98G, and MCF-7 cells were cultured in DMEM medium (Life Technologies, CA) supplemented with 10% FBS, penicillin and streptomycin. DU145 cells were cultured in RPMI 1640 medium with 10% FBS and antibiotics.

### Plasmids

The following constructs were obtained from Addgene: Flag-Smurf1, Flag-Smurf2, Flag-WWP1, Flag-WWP2, Flag-Nedd4-1, Flag-NEDL-1 and Flag-ITCH constructs were obtained from Addgene. The following plasmids were previously described: shGFP, shSmurf1, His-Ub, Smurf1 wild-type and C725A [28], HA-Akt1, HA-Myr-Akt1, HA-Myr-Akt1 KM, HA-Akt2, HA-Myr-Akt2, shAkt1, shAkt2 and Flag-Skp2, [32] Flag-DAB2IP S847A and S847D [20], Flag-DAB2IP and shDAB2IP [16]. Smurf1 and DAB2IP deletion mutants where generated by standard PCR techniques. Smurf1 T145A, T145E, and T145E/148D were generated using Quick-change site directed mutagenesis.

### Cell transfection and viral transduction procedures

For cell transfection, 5 × 10^5^ HeLa or 293T cells were seeded in 100-mm plates and transfected using Lipofectamine (Invitrogen) in OptiMEM medium (Invitrogen) for 48 hours according to the manufacturer's instructions. For viral transduction experiments, 6 × 10^5^ HEK 293T cells were seeded in 60-mm dishes and cotransfected the next day with each lentivirus or retrovirus vector, along with helper plasmids (i.e., gag-pol and VSV-G were used for lentiviral infections). Media with progeny virus from transfected cells was collected at 24 and 48 hours, and then filtered with 0.45-μm filters (Millipore). HeLa, DU145, T98G, and MCF-7 cells were infected with a 1:2 dilution of progeny virus in growth medium with 8 μg/mL polybrene (Sigma-Aldrich). 24 hours post infection, the cells were selected with 1 μg/ml puromycin (Sigma-Aldrich) for 72 hours to eliminate the uninfected cells before collecting lysates for subsequent biochemical assays. Knockdown or overexpression in the transduced cells was confirmed by western blot analysis.

### Antibodies and reagents

The following antibodies were used for this study. Smurf1 (2174), R/KxR/KxxpST (10001) Akt2 (3063), RxRxxpS/T (9614), pERK (4370), ERK (4905), pS473-Akt (4501), and Akt (4691) were from Cell Signaling Technology. c-Myc 9E10 (sc-40), and HA Y-11 (sc-805) were from Santa Cruz Biotechnology. α-Tubulin (T-5168), Vinculin (V-4505), polyclonal Flag (F-2425), monoclonal Flag (F-3165), HA agarose beads (A-2095), peroxidase-conjugated α-mouse secondary antibody (A-4416) and peroxidase-conjugated α-rabbit secondary antibody (A-4914) were from Sigma. GFP (632380) was from Invitrogen. DAB2IP antibody was previously described [16].

### Immunoprecipitation and western blotting

Cells were lysed in EBC-lysis buffer (50 mM Tris, pH 8.0, 120 mM NaCl, and 0.5% NP-40) supplemented with protease inhibitors (Complete Mini; Roche) and phosphatase inhibitors (phosphatase inhibitor cocktail set I and II; EMD Millipore). The protein concentrations of the lysates were measured using a protein assay reagent (Bio-Rad Laboratories, CA) on a DU-800 spectrophotometer (Beckman Coulter). The lysate samples were then resolved by SDS-PAGE and immunoblotted with the indicated antibodies. For immunoprecipitation assays, 20 hrs post transfection, cells were treated with 10 μM MG132 overnight before harvesting for immunoprecipitation. 1 mg of protein lysates were incubated with the appropriate antibodies (1–2 μg) overnight at 4°C, followed by addition of carrier beads. Immunocomplexes were washed five times with NETN buffer (20 mM Tris, pH 8.0, 100 mM NaCl, 1 mM EDTA, and 0.5% NP-40) before being resolved by SDS-PAGE and immunoblotted with indicated antibodies. Ubiquitination assays were performed as previously described [28].

### Colony formation assays

MCF-7 and DU145 were plated in 6-well culture dishes (BD) at a density of 300 cells/well and allowed to grow undisturbed for 9 days. Cells were stained with crystal violet on the plates and counted.

### Soft agar assay

MCF-7 cells were plated in 2% low melting point agar was prepared and mixed with DMEM to make 0.4% and 0.8% agar at 50°C. 2 ml 0.8% agar was added in the bottom of the 6-well plate. 1 × 10^4^ cells/well was mixed with 2 ml 0.4% agar and added on the top of 0.8% agar. Cells were allowed to grow for 15 days. Cells were stained with 1 mg/ml indonitrotetrazolium chloride (Sigma) on the plates and counted.

### *In vitro* scratch assay

MCF-7 and DU145 cells were plated in 60 mm dish. The cell monolayer was scraped in a straight line with a P200 pipet tip. Photographs of the scratch were taken at 0 h and 24 h. Gap width at 0 h was set to 1. Gap width analysis was performed with Image J. Measurements were taken at multiple defined sites (> 6) along the scratch. Each scratch was given an average of all measurements. Data are expressed as the average of three independent experiments.

### Statistical analysis

Student *t* tests was used to evaluate significance between groups all other data, and *p*-values indicated. Error bars represent standard deviation. *p < 0.05, **p < 0.005.

## SUPPLEMENTARY MATERIALS FIGURES


