# Supplementary material for: Smurf1 regulation of DAB2IP controls cell proliferation and migration
Source: Oncotarget. 2016 Mar 27;7(18):26057–69. doi: 10.18632/oncotarget.8424 (PMC5041964; doi:10.18632/oncotarget.8424)
Supplement: Supplementary file 1 [file oncotarget-07-26057-s001.pdf]

Smurf1 regulation of DAB2IP controls cell proliferation and migration

Supplementary Materials

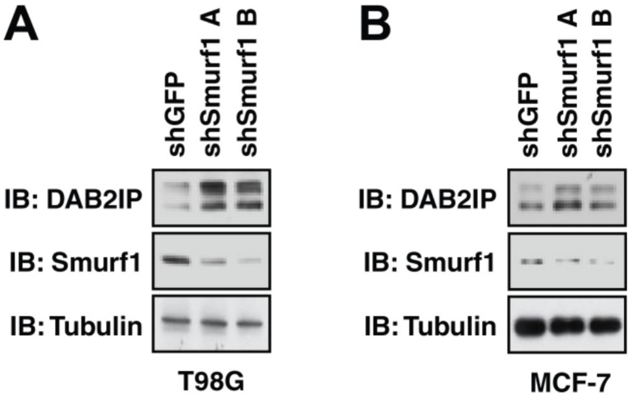

Supplementary Figure S1: Depletion of endogenous Smurf1 induces DAB2IP protein levels. T98G (**A**) and MCF-7 (**B**) cells were infected with virus encoding shRNA against GFP and Smurf1. Following selection of infected cells, whole cell lysates were prepared for western blot analysis with antibodies against DAB2IP, Smurf1 and Tubulin.

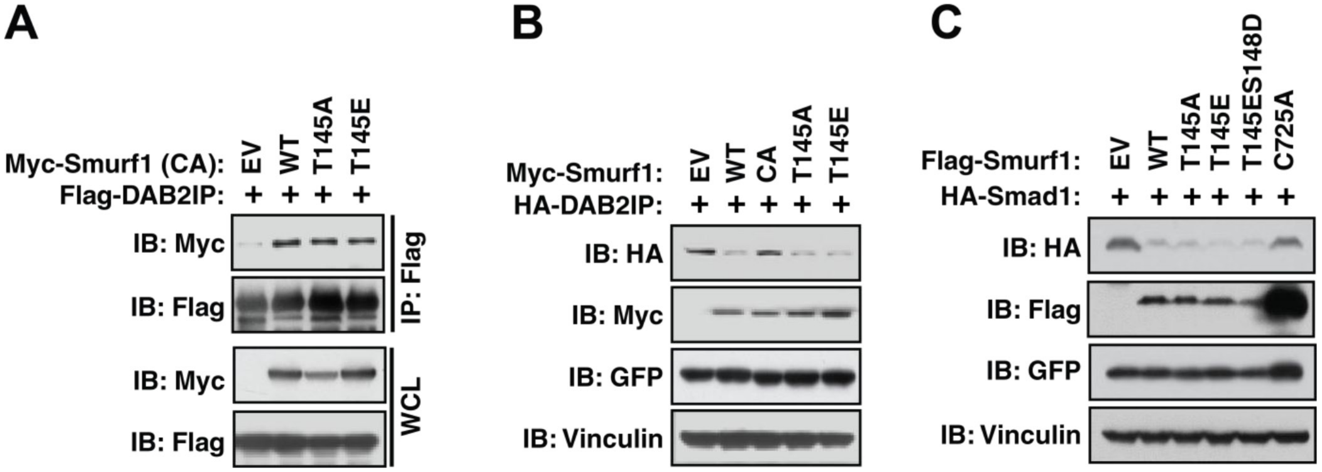

Supplementary Figure S2: Phosphorylation of Smurf1 does not control the interaction with, or direct degradation of, DAB2IP or Smad1. (**A**) 293T cells transfected with Flag-DAB2IP and either control vector, wild-type, T145A, or T145E Myc-Smurf1 C725A (CA) were immunoprecipitated with anti-Flag, and western blotted with antibodies against Myc and Flag. (**B**) 293T cells transfected with HA-DAB2IP and either control vector, wild-type, C725A, T145A, or T145E Myc-Smurf1. Whole cell lysates were prepared for western blot analysis with antibodies against HA, Myc, GFP, and Vinculin. (**C**) 293T cells transfected with HA-Smad1 with control vector, wild-type, C725A, T145A, or T145E, T145E/T148D Flag-Smurf1. Whole cell lysates were prepared for western blot analysis with antibodies against HA, Flag, GFP, and Vinculin.
